# Effects of levels of purity of spores of two *Bacillus* species on their resistance, germination, and core CaDPA and water content

**DOI:** 10.1128/aem.02259-25

**Published:** 2026-06-16

**Authors:** Faith Ye, Dhruv Suryadevara, William J. Bannon, Uttej Kollu, Zaara Khan, Evelyn Cohen, Anita Agrawal, Esther Siw, Adhiti Parupali, Vedant Kansara, George Korza, James Wicander, Vijay K. Juneja, Peter Setlow

**Affiliations:** 1 Department of Molecular Biology and Biophysics, UConn Health 705913, Farmington, Connecticut, USA; 2 U.S. Department of Agriculture, Agricultural Research Service, Eastern Regional Research Center 17123, Wyndmoor, Pennsylvania, USA; Anses, Maisons-Alfort Laboratory for Food Safety, Maisons-Alfort, France

**Keywords:** spore resistance, spore purity, *Bacillus subtilis*, *Bacillus cereus*, spore germination

## Abstract

Previous work (V. K. Juneja, M. Osoria, E. G. Altuntas, N. K. Taneja, et al., Food Res Int 177:113904, 2024, https://doi.org/10.1016/j.foodres.2023.113904) showed that less well-purified spores of *Bacillus cereus and Bacillus subtilis* were more wet heat resistant than more well-purified spores. Extending the previous work, the current work used (i) *B. subtilis* spores identical to the one used previously but lacking a plasmid providing kanamycin resistance and (ii) spores of a different *B. cereus* strain, and with spores made on both plates and in liquid. There were larger differences in spores’ levels of purity in the new work than previously, and differences in spore purity were visible in micrographs of spore preparations purified differently. With spores made on plates, the least purified *B. subtilis* and *B. cereus* spores were more wet heat resistant than those purified further, but less so with spores prepared in liquid. These findings suggest that it may be important to consider the purity of spores prepared as indicators for sterilization assurance, especially since large-scale spore preparations are often not extensively purified. However, resistance to UV radiation, hydrogen peroxide, or sodium hypochlorite was relatively similar with the *B. cereus* or *B. subtilis* spores purified to different degrees. Thus, these methods for spore inactivation appear not be affected significantly by spore purity. Spores’ purity also had minimal effects on rates of germinant-receptor (GR) dependent spore germination with physiological germinants, or with dodecylamine that opens a channel protein leading to release of spore cores’ large pool of Ca-dipicolinic acid (CaDPA). In addition, contents of water in cores of *B. cereus* or *B. subtilis* spores purified to different degrees were almost identical, as were CaDPA levels. All these results do not yet allow obvious assignment of causes for the higher wet heat resistance of cruder *B. subtilis* or *B. cereus* spores. However, it was notable that levels of soluble protein in suspensions of spores purified differently were the highest in the crude spores and lowest in the most highly purified spores.

**IMPORTANCE:**

Spores of many Bacillota give rise to cells and spores, some of which cause food spoilage, food poisoning, and serious human disease. Consequently, there is applied interest in spore killing, something that is difficult because of spores’ resistance to killing regimens including by wet heat, radiation, and toxic chemicals and the many causes of this extreme resistance. The current work shows that for spores of two Bacillus species made on plates or in liquid, spore purity plays no role in the ability of spores to germinate or their resistance to agents other than wet heat. However, the least purified *B. subtilis* or *B. cereus* spores, especially those made on plates, were significantly more wet heat resistant than more purified spores. While precise causes of the effects of spore purity on spore wet heat resistance are not clear, this seems to be an additional variable to consider when preparing spores for various uses, including as in sterilization assurance.

## INTRODUCTION

Bacteria of Bacillota grow in different media, but if media no longer support cell growth, many species undergo the process of sporulation (1). In this process, cells stop growing and divide asymmetrically, with the smaller cell, termed the forespore, engulfed by the larger cell, termed the mother cell. Following forespore development, the mother cell lyses releasing the spore into the environment although spore maturation continues after release if they are held at 37°C (1–4). The released spores are metabolically dormant survival forms that have little if any ATP and are resistant to a variety of agents including wet and dry heat, UV and γ-radiation, desiccation, high pressures, and a host of toxic chemicals including acids, bases, and alkylating and oxidizing agents (5–7). Notably, spores are so dormant and resistant that one report found that spores can survive for many hundreds of years (8).

Dormant Bacillota spores are present in all environments, including soil, water, and the atmosphere (9) and, thus, are in constant contact with humans and animals (9). If these spores remained dormant, this would not be a problem, but they can return to life in germination and grow if conditions are conducive for growth. Importantly, the growing cells of some spore formers can cause food spoilage, food poisoning, uncontrolled diarrhea, infectious disease (*B. anthracis*) plus a variety of intoxications from species such as *Clostridium botulinum* and *Clostridium tetani* (1). Because of spores’ dormancy and resistance, their ubiquity in the environment, and the possibility for bad outcomes if spores germinate and generate growing cells, there has long been interest in how to efficiently and completely kill spores before they can germinate, grow, and cause adverse effects (1, 6, 7).

Many factors influence choices of methods for spore killing including cost, efficiency, possible adverse effects to individuals and the environment, or material, including foodstuffs in the samples to be treated and the pH and temperature (10–12). The latter is of special concern, as if foodstuffs are treated, toxic chemicals cannot be used and treatments such as wet heat must not only kill spores but also cause damage making the food unsalable. One way to assess conditions giving effective spore killing is to use spores of various strains, efficient killing of which has been previously studied. Commonly used strains include spores of *Bacillus atrophaeus, Bacillus pumilus,* or *Clostridium botulinum,* all with relatively similar wet heat resistance (when measured as in this work as the percentage of spore killing at 90–93°C as a function of time) to that of *B. subtilis* spores, and spores of *Geobacillus stearothermophilus*, a thermophile whose spores are more wet heat resistant than those of *B. subtilis* (13–15). Reproducible killing of these spores requires that sporulation variables affecting spore resistance properties be carefully controlled, as sporulation parameters such as temperature, pH, water activity, and ion contents can affect spore resistance, while sporulation medium richness can affect spore germination rates (16, 17). The presence of spore-specific DNA-binding proteins and levels of various integral and peripheral inner membrane (IM) protein homologs also affect spore resistance to many treatments (6, 18–21). *B. subtilis* spores’ resistance to wet heat and some chemicals is also greatly increased the longer spores are kept in sporulation conditions, most notably at 37°C on agar plates (3, 22–24). This latter effect may be due to the maturation of spores after their release from sporangia, at least in part, by increased crosslinking of spore coat proteins (2, 22, 23). Another variable in spore preparation that might modulate spore resistance is the level of purity of the spores to be used. Notably, procedures for purification of spores that have been harvested and kept in water at 4°C vary widely in published reports (25). These range from (i) brief heat treatment to kill remaining growing cells plus one or two centrifugations to remove soluble contaminants; (ii) 4–5 centrifugations and water washing over several days to remove soluble material, unsporulated cells, germinated spores and debris generated by mother cell lysis; (iii) as in (ii) but intermittent sonication for ~3 min over 2 to 3 days to disrupt and disperse mother cell debris such as cell wall and nucleic acid aggregates; and (iv) as in (iii) followed by centrifugation through a high-density solution of Nycodenz (also called Histodenz) in which dormant spores pellet and growing cells, germinated spores, and cell debris float, with water washes by centrifugation to remove the Nycodenz. Note that with spores of species such as *B. cereus* that have an outermost exosporium layer, sonication is not used as it can cause exosporium loss, likely with effects on spore properties.

There are several reasons for concern that spores’ purity might affect their resistance properties, whether to radiation, reactive chemicals, or wet heat as follows. With UV radiation, mother cell lysis releases UV-adsorbing nucleic acids as well as proteins (1). Such impurities could absorb and/or shield UV and, thus, reduce spores’ radiation dose, and if so, minimally purified spores could appear more UV resistant. For reactive chemicals that cause oxidation or alkylation damage, impurities in spore preparations might react with and neutralize such chemicals, again increasing apparent spore resistance. It is more difficult to predict effects of contaminants in spore preparations on spore wet heat resistance, but perhaps impurities might increase this by forming aggregates or shielding spores in some fashion. Indeed, there are reports suggesting that the presence of clumps of spores can increase spores’ wet heat resistance (26).

Notably, a recent report indicated that less pure spores of several *Bacillus* species prepared on plates had higher wet heat resistance than more purified spores also prepared on plates (27). However, very impure spores given only a heat treatment and centrifuged a few times were not tested, nor was heat resistance to spores made in liquid or resistance to any other sporicidal agent. To more thoroughly examine the effects of purity on spore resistance, not only to wet heat but also to UV radiation and the chemicals hydrogen peroxide and hypochlorite, we have used spores prepared either on plates or in liquid that were, in part, less well purified than previously, but otherwise purified similarly (27). We also measured (i) the germination of variously purified spores with several germinants, both germinant receptor (GR)-dependent and a GR-independent germinant, as spores’ ability to germinate and then outgrow into growing cells is crucial for spores to cause adverse effects (1); (ii) compared the spore core’s levels of water and the 1:1 complex of Ca^2+^ and dipicolinic acid (CaDPA) in spores purified differently, as these two parameters have major influences on some spore resistance properties (3); and (iii) measured the levels of soluble protein in spore preps purified differently, as there are reports that the presence of soluble protein in spore preps can increase spore wet heat resistance (28, 29).

## MATERIALS AND METHODS

### Bacterial strains and spore preparation and purification

Two *Bacillus* species were used in this work, *B. subtilis* PS832, a laboratory 168 strain almost identical to the one used previously (27), but lacking the plasmid providing kanamycin resistance, and *B. cereus* T, originally obtained from Harlyn Halvorson. The latter strain was used instead of the one used previously (27), as the latter was an emetic strain that would have been problematic for work by high school students in the Setlow lab. Two independent preparations of *B. subtilis* spores were prepared at 37°C on either 10 2×-Schaeffer’s-Glucose (2×SG) agar plates as described previously (27) or in two aliquots of 250 mL of liquid 2xSG medium in a 2 L flask in an air shaker (30). After 3 days (d) at 37°C, the spores in the liquid medium were harvested by centrifugation for 10 min in a F-10S rotor at 18k RCF, the liquid carefully poured off so as not to discard loose sediment, the pellets suspended in 250 mL of cold deionized Milli-Q (MQ) water, the centrifugation repeated, the supernatant fluid discarded, and the pellet suspended in 40 mL 4°C MQ water. After 3 d at 37°C, spores were scraped from plates and suspended in 40 mL of 4°C MQ water. Two 2 mL aliquots of the suspended spores made in liquid or on plates were centrifuged at 17k RCF in a microcentrifuge for 10 min at 23°C, the pellets suspended in 2 mL of MQ water and heated for 30 min in a water bath at 70°C to kill remaining growing or unsporulated cells, the sample cooled on ice, recentrifuged, the supernatant fluid discarded, the pellet suspended in 2 mL of 4°C MQ water, and samples from the same preps were pooled and stored as the crude samples. The 36 mL of unheated spores made in liquid or on plates were centrifuged at 4°C twice/d for 10 min at 18k RCF in an F-10S rotor over 3 d, the supernatant fluid discarded, the pellet suspended in 36 mL of 4°C MQ water, and 10 mL of the suspensions saved as the centrifuged fractions. The remaining 26 mL was further purified over 4 d by sonication in 4°C water using a MISONIX XL-200 Ultrasonic Liquid Processor with the probe inserted at least halfway down in the liquid. Sonications were for 3 min at ~4% of maximum power to disrupt aggregated impurities and facilitate their removal by further centrifugation, with a total of 5–6 brief sonications followed by centrifugation and the supernatant fluid discarded. The final pellet was suspended in 26 mL of 4°C MQ water and 12 mL saved for further analyses as the sonicated fraction. The remaining 14 mL was centrifuged, the pellet suspended in 3 mL water, 200 μL aliquots were layered on 1.8 mL of 50% Nycodenz (Sigma), and samples were centrifuged at ~16 k RCF for 10 min at 4°C in a microcentrifuge. In these conditions, dormant spores pellet while cells, germinated spores, and debris float (25). The supernatant fluid was discarded, the pellet fraction suspended in 4 mL of 4°C MQ water, any Nycodenz removed by four washes with water and the final pellets were suspended in ~10 mL of 4°C MQ water as the Nycodenz fraction. Note that this entire purification regimen had 15–20 water washings of the spores. All saved fractions were stored at 4°C protected from light. Note that spore preparations that are purified using only heat treatment and/or water washing have large amounts of debris readily seen above spore pellets following centrifugation, while this material is largely removed by further purification as described previously (25) and see Results. Spores were used for experiments within 2 months of their storage at 4°C.

**Fig 1:**
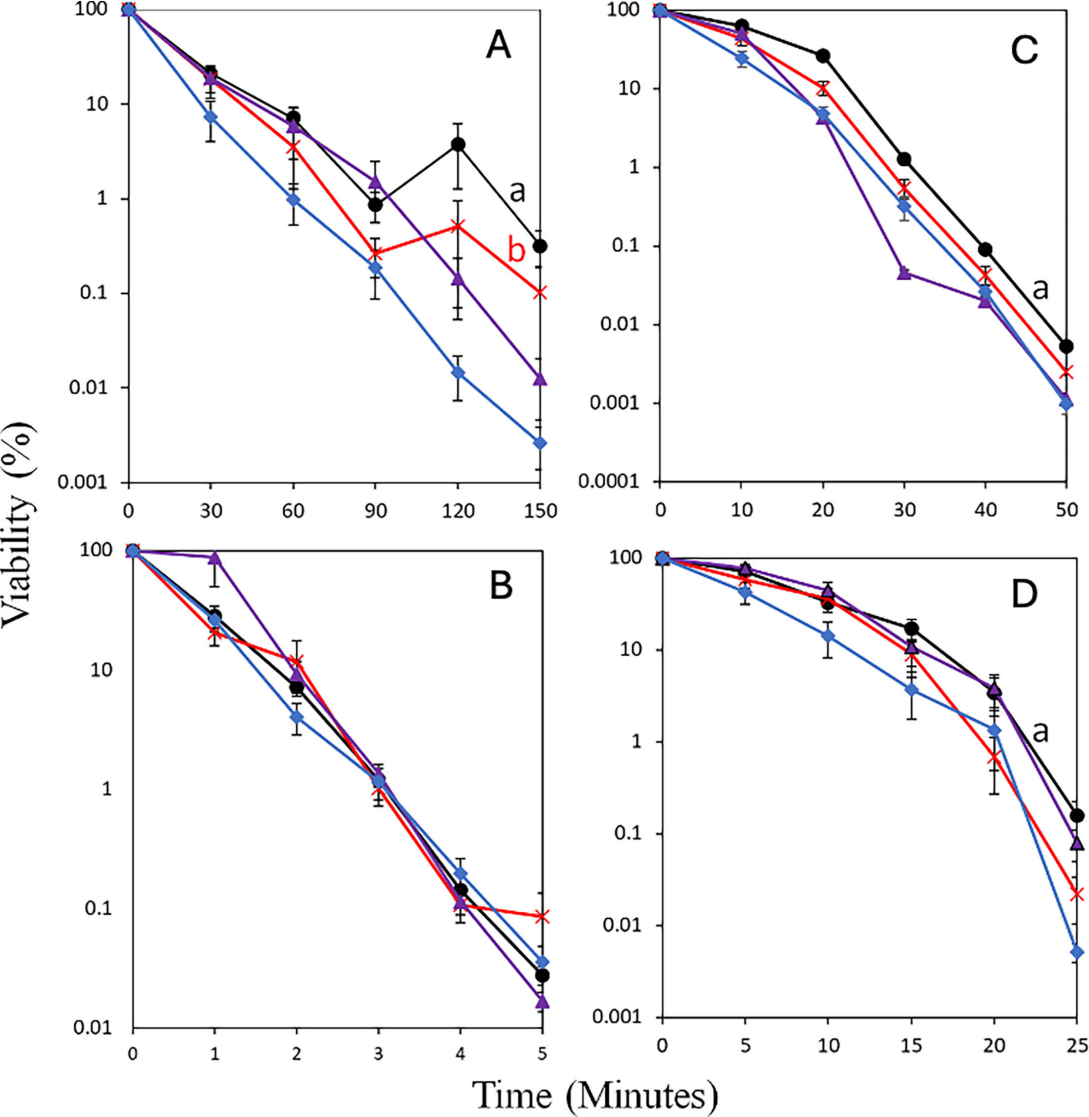
(**A–D**) Resistance properties of *B. subtilis* spores made on plates and purified differently. Spores were prepared on plates and purified differently and treated as described in Materials and Methods with (**A**) wet heat, (**B**) UV radiation, (**C**) hydrogen peroxide, or (**D**) sodium hypochlorite, and spore viability was measured, all as described in Materials and Methods. The data points at each time point represent the log-transformed average viability of at least eight measurements on each spore sample, four on each of two independent isolates with error bars indicating the standard error of the mean (SEM). The a and b above curves denote statistically significant differences (*P* ≤ 0.05) between the Nycodenz sample and the (**A**) crude and (**B**) centrifuged samples. The absence of a letter indicates no significant difference (*P* > 0.05). Symbols for spores of different purity are black line and black filled circle—crude; red line and red cross—centrifuged only; purple line and purple filled triangle—centrifuged and sonicated; blue line and filled blue diamond—run all through Nycodenz treatment.

*B. cereus* spores were prepared as two independent batches of 10 supplemented nutrient broth (SNB) plates at 30°C as described (27) or in liquid SNB medium as described above for *B. subtilis* spores except at 30°C. The spores were harvested after 2 1/2 d of incubation as described above. Further purification of *B. cereus* spores was as described above for *B. subtilis* spores, except that sonication was not used as this can damage spores’ exosporium which is not present in *B. subtilis* spores. These samples were also saved at 4°C protected from light. Note that four independent preparations of spores of both species were obtained, two made on plates and two in liquid such that there were four independent samples of each purification category for the species examined. It is important to note that spores can accumulate significant changes in their resistance after harvest, but not when held at 4°C (3, 4). Notably, ungerminated individual spores made up >99% of the spores in the Nycodenz purified samples of *B. cereus* and *B. subtilis* spores as seen in bright field micrographs obtained by phase contrast microscopy (Fig. 1A and B). However, the crude, water washed, and sonicated spore preps had significant levels of impurities that could be seen under microscopy (Fig. 1C and D). Notably, levels of germinated spores and growing cells as well as visible debris decreased as spores were given the overall purification regimen as seen in Fig. 1A through D.

### Analysis of spore killing

Spores purified differently were given four sporicidal treatments: wet heat and incubation with hydrogen peroxide, sodium hypochlorite, or UV light. All treatments were carried out with a minimum of two separate experiments for each purified sample. In each experiment, spore viability was measured in duplicate at all time points, and all data for each treatment were averaged. This meant that data points for killing treatments for each purification sample, whether with spores made on plates or in liquid, were averages of at least eight determinations of spore viability at each time tested; with duplicate determinations in each experiment, data points at the same times were averaged, and statistical significances were determined as described in Materials and Methods. Note that the resistance data for all the treatments presented show how fast spores are killed throughout the various time points indicated. The faster the killing indicates less resistance, the slower the killing indicates more resistance. The typical resistance parameter of reduction of initial population by 90% is not used here since the killing was not entirely linear over the various time points.

Conditions for spore treatments were as follows (17, 18, 20, 30–34), with spores incubated at an optical density at 600 nm (OD_600_) of ~1 (~10^8^ spores/mL), generally in 1 mL of treatment solution, but with 3 mL for UV treatment: (i) wet heat at 93°C (*B. subtilis*) or 90°C (*B. cereus*) and 50 μL aliquots taken at various times were added to 450 μL of sterile cold MQ water and diluted 1/10 serially in sterile MQ water up to 10^−6^; (ii) sodium hypochlorite—spores were incubated at 23°C in NaOCl with 2.5% available chlorine, and 50 μL aliquots were taken at various times and added to 450 μL of sterile 1% sodium thiosulfate to inactivate the hypochlorite, samples incubated for 30 min and 50 μL serially diluted up to 10^−6^ in 450 μL sterile MQ water; (iii) hydrogen peroxide—spores were incubated at 23°C in 11% hydrogen peroxide in sterile 50 mM KPO_4_ buffer at pH 7.4 for 10 min, and then 50 μL samples taken at 10 min intervals were diluted in 450 μL of catalase (~5 µg/mL in 50 mM KPO_4_ buffer, pH 7.4), incubated for 10 min to inactivate the hydrogen peroxide and samples then serially diluted 1/10 to 10^−6^ in sterile MQ water; and (iv) UV light—3 mL spores at an OD_600_ of 1 in MQ water were added to a ~2.5 cm diameter sterile plastic dish without a cover that was 27 cm directly below a UV lamp (UVP UVG-11, Analytic Jens, USA) maximally emitting at 254 nm with ~100 μW/cm^2^, and 50 μL samples were taken at various times and serially diluted 10-fold in sterile MQ water to 10^−6^. For dilutions of treated samples, 10 μL aliquots were spotted in duplicate in a grid on LB agar medium plates (21, 22, 27, 34), the spots allowed to dry in, plates incubated at 30°C overnight and then at 37°C until no more colonies appeared, and colonies were counted.

### Analysis of spore germination

After heat activation (35) at 70°C for 30 min for spores at an optical density at 600 nm (OD_600_) of 5–10 and then cooling, spores at an OD_600_ of 0.5 in 200 μL of 25 mM K-Hepes buffer pH 7.4 plus 50 μM TbCl_3_ were germinated at 37°C with the GR germinants 10 mM L-alanine (*B. cereus*) or 10 mM L-valine (*B. subtilis*). Germination in these experiments was monitored by duplicate measurements of the release of the spore core’s large amount of CaDPA by DPA’s fluorescence with terbium in a multi-well fluorometric plate reader, results were averaged (34, 36), and statistical significances were determined as described in Materials and Methods.

*B. cereus* and *B. subtilis* spores were also germinated with 1 mM dodecylamine which acts not on GRs but opens the spores’ IM CaDPA release channel protein SpoVAC (37). Dodecylamine germination requires no heat activation, but is sensitive to spores’ IM rigidity, and rates of dodecylamine germination decrease markedly in spores with higher IM rigidity (20, 21, 34, 35). This germination was at 40°C in ~4 mL of 25 mM Tris-HCl, pH 8.0, and 1 mM dodecylamine with spores at an OD_600_ of 0.5 (21). Germination was started by spore addition, and at various times, 450 μL was mixed with 50 μL of 500 μM TbCl_3_, and Tb-DPA fluorescence was measured; duplicate values were taken at each time point, and duplicates were averaged (33).

### Statistical analyses

Statistical analyses were performed as described in reference 38 using GraphPad Prism 10 (GraphPad Software, San Diego, CA). For experiments comparing two groups, unpaired Student’s *t*-tests were used. For experiments comparing three or more groups, one-way ANOVA was performed first; when ANOVA indicated significant differences (*P* < 0.05), pairwise unpaired *t*-tests were conducted. For data with large dynamic ranges (colony-forming units, relative fluorescence units), log transformation was applied prior to statistical analysis. For percent viable data, no transformation was applied. Statistical significance was set at *P* < 0.05, and significant differences are indicated by letters a or b above lines in figures. All experiments included *n* = 2 biological replicates per group.

### Measurement of spore cores’ CaDPA and water content

Spore cores’ CaDPA content was determined as described (39–42), and the relative core water contents in spores of various purities were determined by parallel density gradient centrifugations of intact spores in a Nycodenz gradient and comparing the banding positions of the spores in the gradients as described (39, 40). For this equilibrium density gradient centrifugation, 2 ODs of spores in water were first pelleted and then suspended in 100 μL of 30% Nycodenz. Samples were left on ice for 1 h and then layered on a 2 mL density gradient of 76% to 40% Nycodenz in steps of 2% in Beckman ultraclear tubes (11 × 34 mm). Every other step was marked on the tubes. The tubes were centrifuged at 20°C for 45 min at 14k RCF in a TLS55 swinging bucket rotor in a Beckman TL-100 ultracentrifuge with deceleration set to 8. When done, the position of spore bands was determined using the marks on the tubes.

### Assessments of spore purity by microscopy and analyses of soluble protein in spore preparations

To directly assess the purity of spores purified to different degrees, 10 μL of spore preps at an OD_600_ nm of ~10 was spotted on a microscope slide, and the slides were photographed under bright field microscopy (Fig. 7 and 8). Germinated spores are denoted by white arrows, growing cells by gray arrows and clumps of debris are indicated by black arrows.

For analyses of soluble protein in spore preps, protein levels were determined as follows: after the final wash in each purification step, spores obtained from plates and liquid were resuspended in water to OD_600_ = 50. One milliliter was transferred to microfuge tube and centrifuged at 17k RCF for 2 min at room temperature. Supernatants were assayed for total protein concentration using NanoDrop 2000 Protein A280 protocol as described in the user manual. A standard curve was made using bovine serum albumin (BSA) concentration range from 0.01 to 2.00 mg/mL. Values were obtained on all duplicate samples, and the average amounts are given in Table 2.

## RESULTS

### Resistance to various agents of spores of varying purities

**Fig 2:**
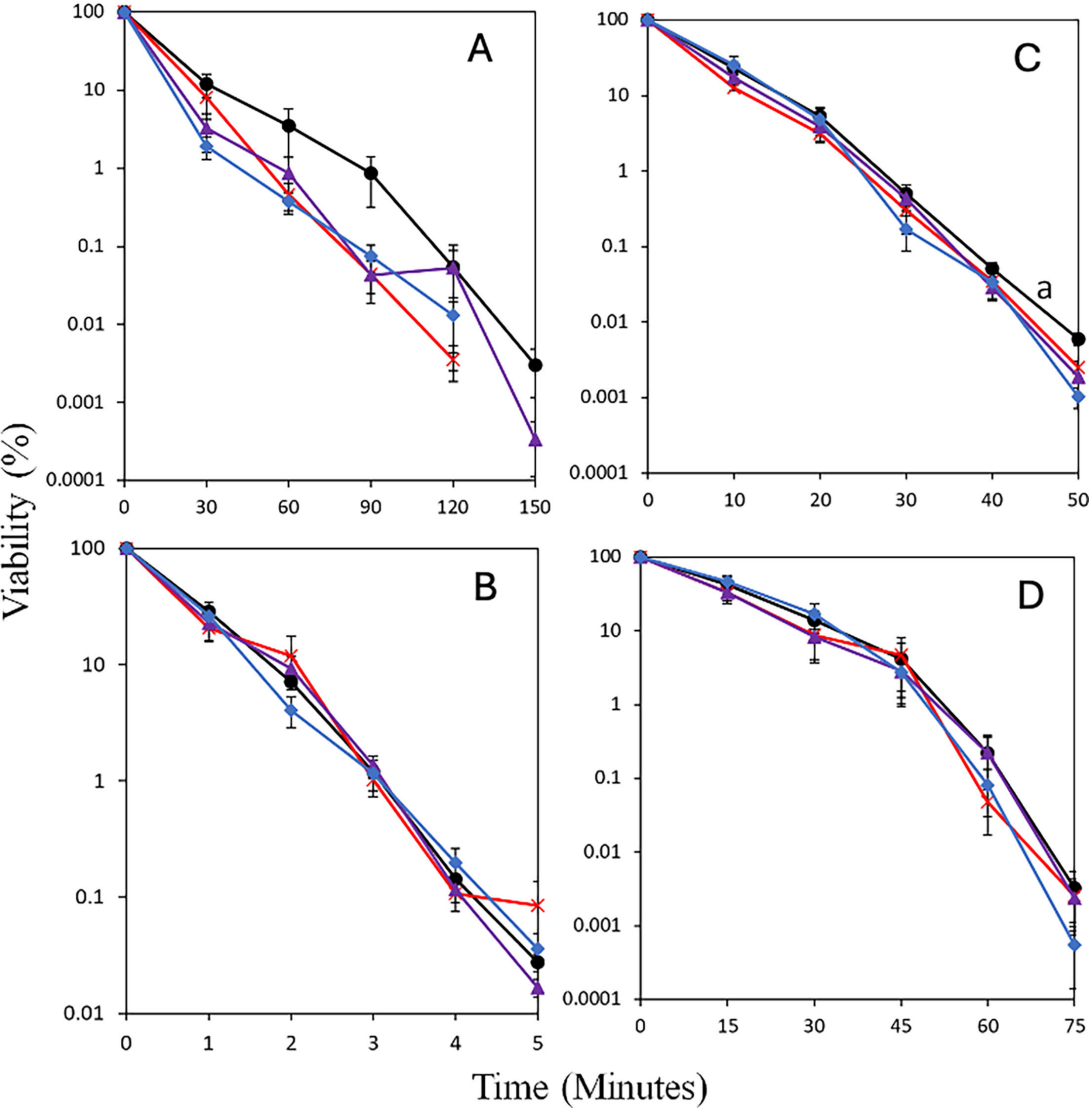
(**A–D**) Resistance properties of *B. subtilis* spores made in liquid and purified differently. Spores were prepared in liquid and purified differently and treated as described in Materials and Methods with (**A**) wet heat, (**B**) UV radiation, (**C**) hydrogen peroxide, or (**D**) sodium hypochlorite, and spore viability was measured, all as described in Materials and Methods. The data points at each time point represent the log-transformed average viability of at least eight measurements on each spore sample, four on each of two independent isolates with error bars indicating the standard error of the mean (SEM). The a and b above curves denote statistically significant differences (*P* ≤ 0.05) between the Nycodenz sample and the (**A**) crude and (**B**) centrifuged samples. Symbols for spores of different purity are black line and black filled circle—crude; red line and red cross—centrifuged only; purple line and purple filled triangle—centrifuged and sonicated; blue line and filled blue diamond—run all through Nycodenz treatment.

**Fig 3:**
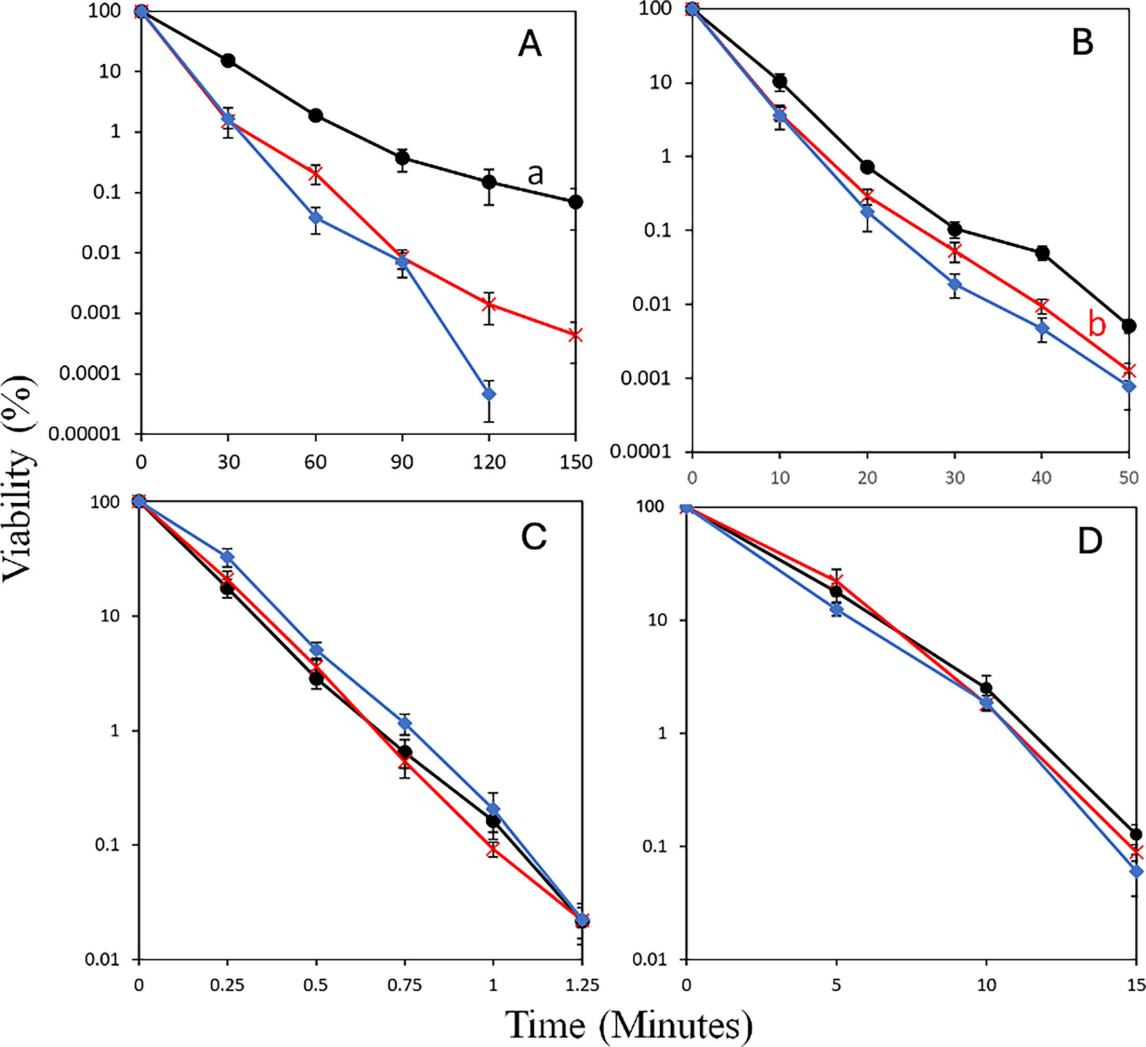
(**A–D**) Resistance properties of *B. cereus* spores made on plates and purified differently. Spores were prepared on plates and purified differently and were treated as described in Materials and Methods with (**A**) wet heat, (**B**) UV radiation, (**C**) hydrogen peroxide, or (**D**) sodium hypochlorite, and spore viability was measured, all as described in Materials and Methods. The data points at each time point represent the log-transformed average viability of at least eight measurements on each spore sample, four on each of two independent isolates with error bars indicating the standard error of the mean (SEM). The letters a and b above curves denote statistically significant differences (*P* ≤ 0.05) between the Nycodenz sample and the (**A**) crude or (**B**) centrifuged samples. Symbols for spores of different purity are black line and black filled circle—crude; red line and red cross—centrifuged only; and blue line and filled blue diamond—run all through Nycodenz treatment.

**Fig 4:**
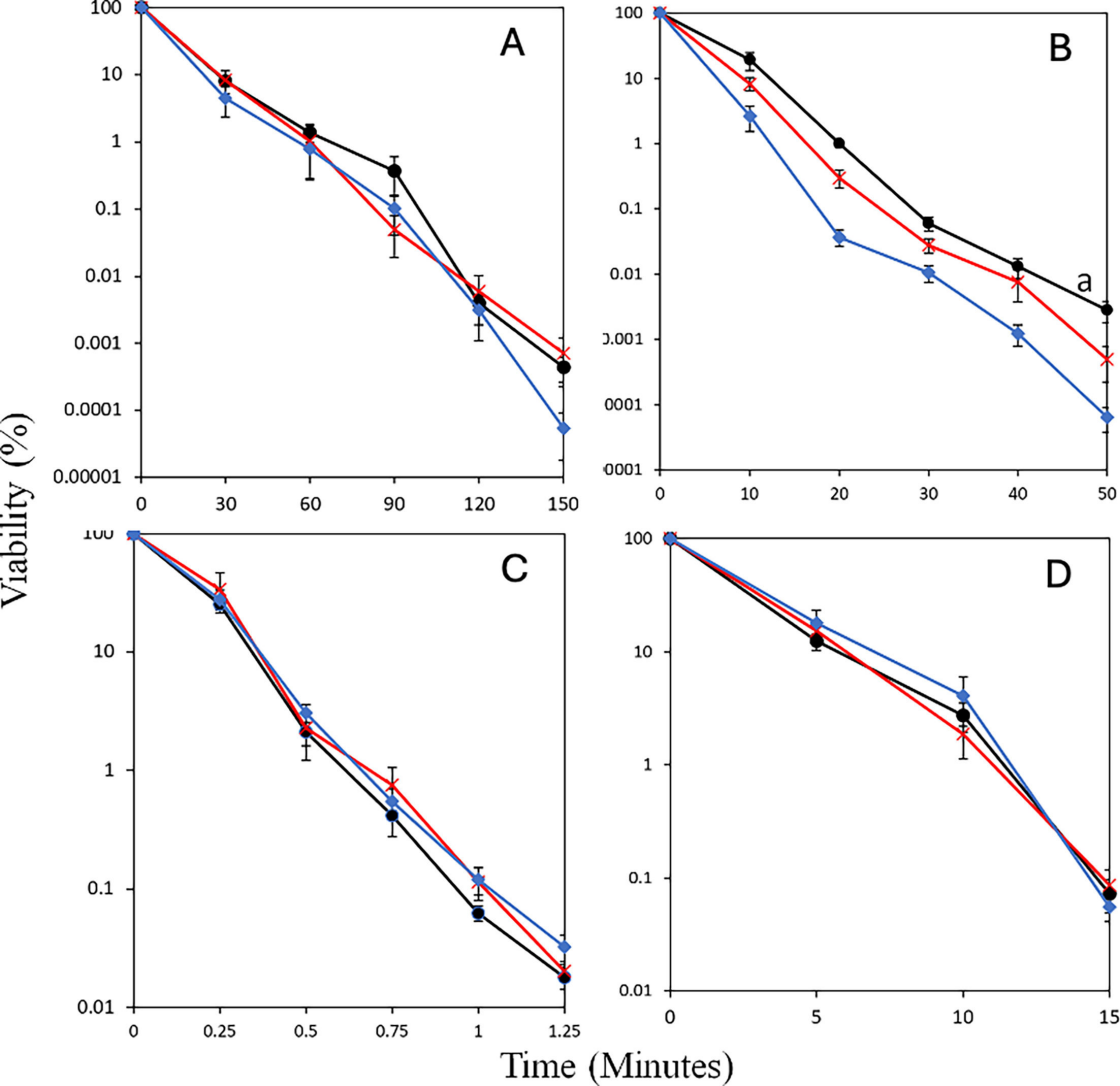
(**A–D**) Resistance properties of *B. cereus* spores made in liquid and purified differently. Spores were prepared in liquid and purified differently and were treated with (**A**) wet heat, (**B**) UV radiation, (**C**) hydrogen peroxide, or (**D**) sodium hypochlorite, and spore viability was measured, all as described in Materials and Methods. The data points at each time point represent the log-transformed average viability of at least eight measurements on each spore sample, four on each of two independent isolates with error bars indicating the standard error of the mean (SEM). The letters a and b above curves denote statistically significant differences (*P* ≤ 0.05) between the Nycodenz sample and the (**A**) crude and (**B**) centrifuged samples. Symbols for spores of different purity are black line and black filled circle—crude, red line and red cross—centrifuged only, and blue line and filled blue diamond—run all through Nycodenz treatment.

*B. subtilis* and *B. cereus* spores of varying purity were exposed to four sporicidal treatments and their resistance determined. The crude *B. subtilis* and *B. cereus* spores made on plates were significantly more wet heat resistant than more purified spores, with wet heat resistance decreasing in the purer spores, while spores made in liquid were less wet heat resistant (Fig. 1A, 2A, 3A, and 4A) as found previously (21, 22). Purer *B. subtilis* or *B. cereus* spores made on plates or in liquid were not significantly less resistant to UV radiation than less well purified spores (Fig. 1B, 2B, 3B and 4B). However, cruder *B. subtilis* and *B. cereus* spores made in liquid or on plates exhibited slightly lower but significant resistance to hydrogen peroxide than spores purified through Nycodenz and again with slight but significantly increased hypochlorite resistance with *B. subtilis* spores prepared on plates (Fig. 1C, 2D, 3C and 4C).

### Effects of spore purity on spore germination

**Fig 5:**
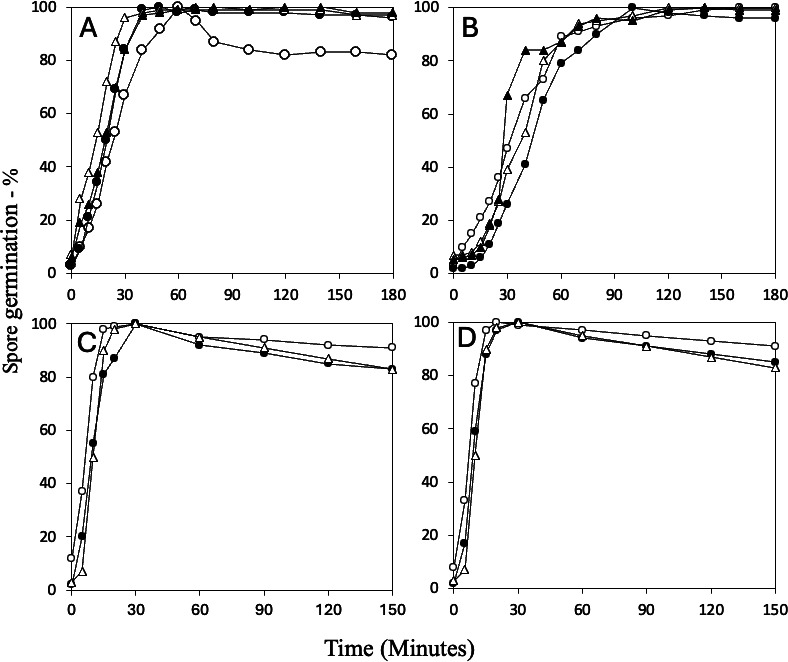
(**A–D**) Germination of *B. subtilis* (**A and B**) and *B. cereus* (**Cand D**) spores prepared in liquid (**A and C**) or on plates (**B and D**) and with different levels of purity. Spores purified to different degrees were heat activated and germinated as described in Materials and Methods with (**A and B**) 10 mM L-valine or (**B and D**) 10 mM L-alanine and percent germination values determined in quadruplicate were averaged. Symbols used for the spores treated are as follows: in panels A and B, open circles, crude; filled circles, centrifuged; open triangles, centrifuged and sonicated; filled triangles, Nycodenz purified; in panels C and D, open circles, crude; filled circles, centrifuged; open triangles, Nycodenz purified. All values shown are averages of two determinations each on two independent spore preparations, and no statistically significant differences (*P* > 0.05) were observed between the germination of spores purified differently.

Since spores can only exert detrimental effects when they germinate and become growing or stationary phase cells, the germination of *B. cereus* and *B. subtilis* spores purified to various degrees was also tested. The germinants used, L-valine for *B. subtilis* spores and L-alanine for *B. cereus* spores, are recognized by specific GRs (3–7). Germinant-GR interaction then triggers CaDPA release from the spore core, and this triggers hydrolysis of the spores’ peptidoglycan cortex around the IM, leading to core water uptake and swelling, resumption of spore metabolism, and ultimately cell growth (33). Notably, *B. subtilis* spores purified to different degrees exhibited no significant differences in L-valine germination, although this was more rapid with spores prepared in liquid (Fig. 5A and B), as found previously (18, 19, 21). Similarly, germination of *B. cereus* spores by L-alanine was essentially identical (*P* > 0.05) with spores purified to different degrees and again faster with spores prepared in liquid (Fig. 5C and D). Previous work has indicated that the slower germination of spores prepared on plates is likely due to the more rigid IM in spores prepared on plates (21) although exactly why the IM is more rigid is not clear.

**Fig 6:**
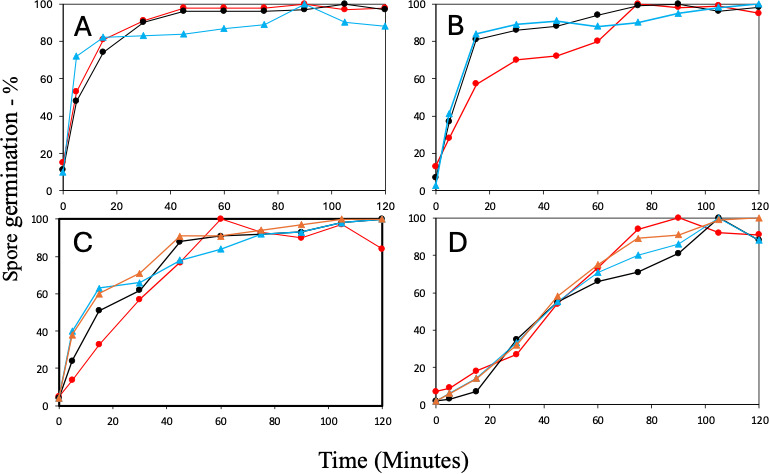
(**A–D**) Dodecylamine germination of *B. subtilis* or *B. cereus* spores made in liquid or on plates and with different purity. Differently purified (**A and B**) *B. cereus* or (**C and D**) *B. subtilus* spores made in liquid (**A and C**) or on plates (**B and D**) were germinated with dodecylamine as described in Materials and Methods and values for germination measured by DPA released were averaged. Symbols used for the *B. subtilis* spores treated are black lines and filled circles—crude; red lines and red filled circles—centrifuged; orange line and orange filled triangles—centrifuged and sonicated; and blue lines and blue filled triangles—Nycodenz purified. For *B. cereus* spores, the symbols used are black lines and filled circles—crude; red lines and red filled circles—centrifuged; and blue lines and blue filled triangles—Nycodenz purified. All values shown are averages of two determinations on each of two spore preparations made in liquid or on plates, and no statistically significant differences (*P* > 0.05) were observed between the germination of spores purified differently.

When the GR-independent germinant dodecylamine was used, germination was much faster with *B. cereus* but was essentially identical (*P* > 0.05) in either *B. cereus* or *B. subtilis* spores with different levels of purity (Fig. 6A and B). These results indicate that spores’ level of purity does not affect spore IM fluidity, as spores with a more fluid IM germinate faster with dodecylamine than in spores with a less fluid IM (18–20).

### Effects of spore purity on core CaDPA and water content

Since spore cores’ levels of CaDPA and water can affect spore resistance, in particular to wet heat (3), these levels were also determined in spores with different levels of purity. Notably, relative CaDPA levels were essentially identical in the spores of either *B. cereus* or *B. subtilis* with different levels of purity, as was also the case when relative core water contents were measured in spores by the banding positions of spores in Nycodenz density gradients (Table 1).

**TABLE 1:** Relative CaDPA and water contents in cores of spores purified to different levels^*a*^

Parameter	*B. subtilis*			*B. cereus*		
	Cr	Son	Nyco	Cr	Cfg	Nyco
CaDPA content^*b*^	96	100	98	98	97	100
Intact spores-Nyc%^*c*^	60	62	62	56	57	57
Decoated spores-Nyc%^*c*^	64	66	66	58	58	58

^*a*^ Core contents of CaDPA and water in spores of two species purified to different degrees were determined as described in Materials and Methods. Abbreviations denote the different levels of spore purity as described in Materials and Methods: Cr, Crude; Son, sonicated; Cfg, centrifuged; Nyco, Nycodenz purification. All values are ±2%.

^*b*^ Values are the levels of CaDPA in the spore core relative to the highest level that was set as 100%.

^*c*^ Values are the percent Nycodenz (Nyc) banding positions of intact or decoated spores in Nycodenz gradients.

### Protein levels in supernatant of different spore preparations

Analysis of soluble proteins in the supernatant of the final washes showed quantifiable levels in the crude and centrifuged fractions but none detected in the sonicated or Nycodenz samples (Table 2).

**TABLE 2:** Total protein levels (mg/mL) in final wash of spores purified to different levels^*a*^

Preparation	*B. subtilis*				*B. cereus*		
	Cr	Cfg	Son	Nyco	Cr	Cfg	Nyco
Plates	0.33 ± 0.03	0.08 ± 0.03	<0.01	<0.01	0.46 ± 0.05	0.19 ± 0.10	<0.01
Liquid	0.38 ± 0.08	0.11 ± 0.03	<0.01	<0.01	0.75 ± 0.10	0.21 ± 0.03	<0.01

^*a*^ Protein concentration in final supernatant wash of spores of two species purified to different degrees was determined as described in Materials and Methods. Abbreviations denote the different levels of spore purity as described in Materials and Methods: Cr, Crude; Son, sonicated; Cfg, centrifuged; Nyco, Nycodenz purification.

## DISCUSSION

One conclusion from the work in this communication is that differences in spores’ purity have no detectable effects on spore germination by GR-dependent germinants. It is known that *B. subtilis* sporulation conditions, in particular the sporulation medium richness, can affect the germination ability of resultant spores, with spores made in richer media having increased GR levels, and, thus, germinate more rapidly (14). While we have not looked at GR levels in spores at various degrees of purity, the similar germination rates for the differently purified spores of two species and via several GRs indicate that spore purity does not significantly affect GR levels or function.

**Fig 7:**
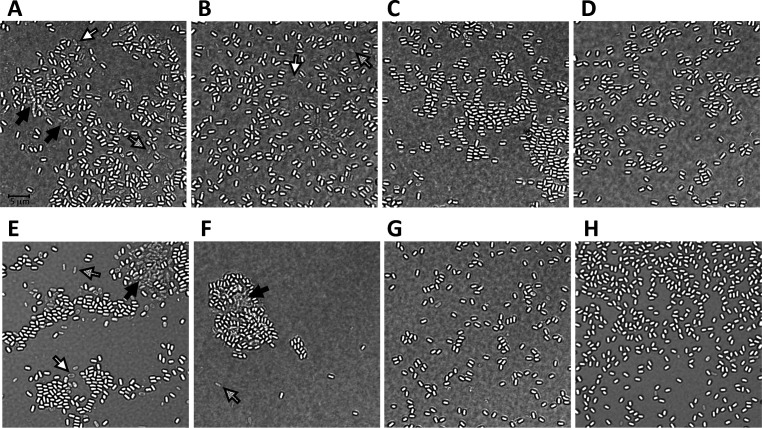
(**A–H**) Brightfield images of *B. subtilis* spores made on plates: (**A**) crude fraction, (**B**) centrifuged fraction, (**C**) sonicated fraction, and (**D**) purified with Nycodenz; or spores made in liquid: (**E**) crude fraction, (**F**) centrifuged fraction, (**G**) sonicated fraction, and (**H**) purified through Nycodenz, all as described in Materials and Methods. Images were obtained on a Zeiss LSM 880 microscope (63× lens). White arrows indicate growing cells, black arrows indicate clumps of debris, gray arrows indicate germinated spores. A 5 μm scale bar is shown in panel A.

**Fig 8:**
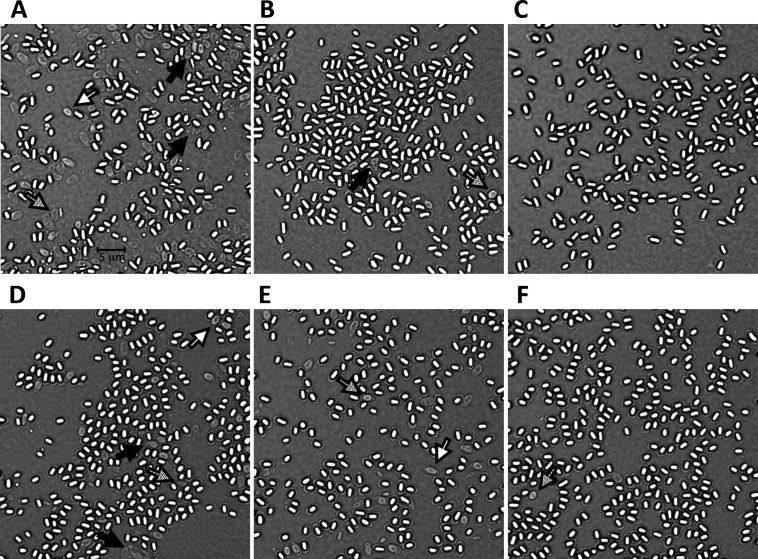
(**A–D**) Brightfield images of *B. cereus* spores made on plates: (**A**) crude fraction, (**B**) centrifuged fraction, and (**C**) purified with Nycodenz or spores made in liquid: (**D**) crude fraction, (**E**) centrifuged fraction, and (**F**) purified with Nycodenz, all as described in Materials and Methods. Images were obtained on a Zeiss LSM 880 microscope (63× lens). White arrows indicate growing cells, black arrows indicate debris impurities, gray arrows indicate germinated spores. A 5 μm scale bar is shown in panel **A**.

A second conclusion is that the purity of the spores used here, whether made in liquid or on plates, had no major effects on *B. subtilis* and *B. cereus* spore resistance to UV radiation, and effects on spore hydrogen peroxide resistance, while significant, were small, and there were no significant effects of *B. subtilis* spore’s purity on resistance to hypochlorite. All this plus the much lower effects of spore preparation in liquid on spore wet heat resistance suggests that preparation of spores on plates or in liquid should be evaluated when making spores to be used as indicator strains. However, there remains the situation with the wet heat resistance of *B. subtilis* and *B. cereus* spores which was highest in the least well-purified spores and decreased as spores were purified further, as found previously (27). Note that the latter report did not examine spores akin to the crude spores used in the current work, and thus the current work found even larger effects than previously (27). A major question then raised by the current and previous work is why spore wet heat resistance is so much higher in the cruder *B. subtilis* and *B. cereus* spores made on plates. Multiple factors affect spores’ wet heat resistance as follows. (i) The spores’ core water content, with lower values associated with higher wet heat resistance, and CaDPA accumulation and compression of the spore core are major factors reducing spore core water content late in sporulation (3, 6, 7). However, there were no significant differences in either CaDPA or core water content in spores of different purity. (ii) Saturation of core DNA with small acid-soluble proteins (SASP) that also protect spore DNA against UV, and hydrogen peroxide, but not hypochlorite (30). However, the nearly identical resistance of *B. subtilis* and *B. cereus* spores of different purity to UV radiation indicates that SASP protection of DNA has not been greatly affected in spores of different purity. (iii) Differences in IM fluidity can also affect spore wet heat resistance, with a more rigid IM leading to higher wet heat resistance (15–17). However, the nearly identical dodecylamine germination rates of *B. subtilis* and *B. cereus* spores with different levels of purity (Fig. 6) are consistent with their being minimal differences in the IM fluidity in these spores (15, 17), which would rule out changes in this spore property altering changes in wet heat resistance of spores of different purity. While relatively minor differences in osmotic pressure/ionic strength are known to influence spore wet heat resistance, this is unlikely in this situation since all water used was MQ ultrapure water and there were two MQ water washes of harvested spores prior to obtaining the crude fraction. However, the protein remaining in crude spores, with minimal protein in the less well- purified spores (Table 2), would itself alter the osmotic pressure in the crude spores and contribute to spore wet heat resistance. One striking difference between the cruder and more purified spores was that while spores made in liquid or on plates and purified through Nycodenz were almost all single dormant spores (Fig. 7B, 8B and D), the cruder spores had not only dormant spores, growing cells, and germinated spores but also clumps of debris with dormant spores in the vicinity (black arrows, Fig. 7A and C, 8A and C). Perhaps the presence of the debris and soluble protein (Table 2) insulates the dormant spores protecting them from wet heat, but further work will be needed to prove this. Conversely, perhaps there is another factor modulating spore wet heat resistance yet to be discovered. Indeed, two new major factors modulating spore wet heat resistance were discovered only very recently (17, 18), indicating that spores do not easily give up their secrets, and thus, there is still work to be done to fully understand all mechanisms of spore wet heat resistance. Notably, this may ultimately be of benefit to the food industry.
